# The Relationship between the Gut Microbiome and Metformin as a Key for Treating Type 2 Diabetes Mellitus

**DOI:** 10.3390/ijms22073566

**Published:** 2021-03-30

**Authors:** Chae Bin Lee, Soon Uk Chae, Seong Jun Jo, Ui Min Jerng, Soo Kyung Bae

**Affiliations:** 1 College of Pharmacy and Integrated Research Institute of Pharmaceutical Sciences, The Catholic University of Korea, Bucheon 14662, Korea; aribri727@catholic.ac.kr (C.B.L.); zldtnseo@catholic.ac.kr (S.U.C.); seongjun6734@catholic.ac.kr (S.J.J.); 2 Department of Internal Medicine, College of Korean Medicine, Sangji University, Wonju 26339, Korea; healmind@paran.com

**Keywords:** gut microbiome, type 2 diabetes mellitus, metformin, dysbiosis

## Abstract

Metformin is the first-line pharmacotherapy for treating type 2 diabetes mellitus (T2DM); however, its mechanism of modulating glucose metabolism is elusive. Recent advances have identified the gut as a potential target of metformin. As patients with metabolic disorders exhibit dysbiosis, the gut microbiome has garnered interest as a potential target for metabolic disease. Henceforth, studies have focused on unraveling the relationship of metabolic disorders with the human gut microbiome. According to various metagenome studies, gut dysbiosis is evident in T2DM patients. Besides this, alterations in the gut microbiome were also observed in the metformin-treated T2DM patients compared to the non-treated T2DM patients. Thus, several studies on rodents have suggested potential mechanisms interacting with the gut microbiome, including regulation of glucose metabolism, an increase in short-chain fatty acids, strengthening intestinal permeability against lipopolysaccharides, modulating the immune response, and interaction with bile acids. Furthermore, human studies have demonstrated evidence substantiating the hypotheses based on rodent studies. This review discusses the current knowledge of how metformin modulates T2DM with respect to the gut microbiome and discusses the prospect of harnessing this mechanism in treating T2DM.

## 1. Introduction

**Figure 1:**
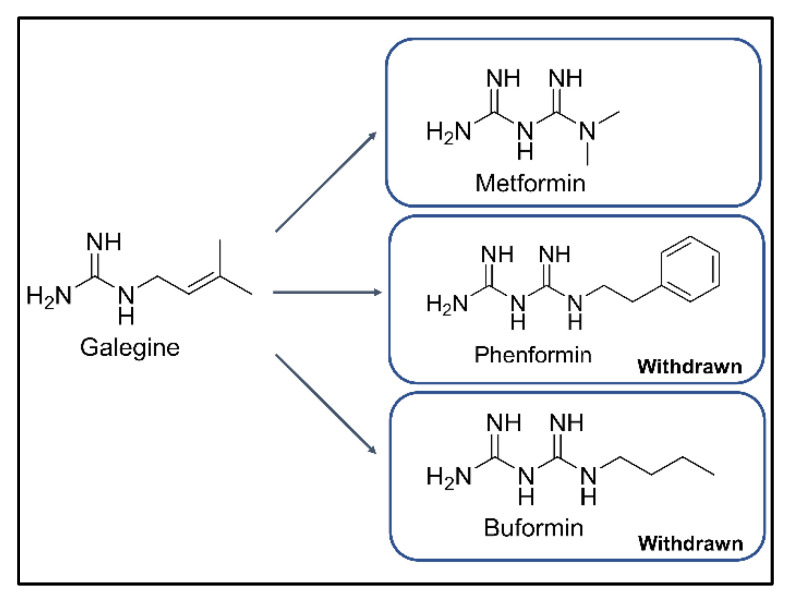
Chemical structures of galegine, metformin, phenformin, and buformin.

Type 2 diabetes mellitus (T2DM) is one of the most common chronic metabolic disorders and is characterized by hyperglycemia resulting from the combination of insulin resistance and inadequate insulin secretion [1,2,3,4]. The number of people with T2DM has drastically increased over the past several decades [1]. Metformin, a biguanide class drug, is recommended by the American Diabetes Association and European Association for the Study of Diabetes as a first-line medicine for the treatment of T2DM [2]. Metformin is a derivative of phenformin and buformin from galegine in *Galega officinalis*, traditionally used to decrease blood sugar and relieve the symptoms of diabetes (Figure 1) [3,4]. Among the three biguanides, phenformin and buformin were withdrawn from the market due to the high frequency of lactic acidosis in the 1970s. However, metformin showed superior safety and better efficacy in the treatment of T2DM [5,6,7,8,9]. These advantages for clinical use have resulted in metformin being widely used for more than 60 years [5]. Metformin does not target a specific pathway or disease mechanism [4]; therefore, studies have aimed to reveal the mechanism of action of metformin related to the treatment of cancer and cardiovascular diseases [1,2,3,4,5]. Metformin exhibits the peak plasma concentrations in 3 h with C_max_ 1.0–1.6 mg/L for dose of 500 mg and approximately 55% of bioavailability ([6] and refences therein). After absorption, metformin is distributed in the liver, kidneys, adrenal glands, and pancreas at about seven-fold higher concentration than that of the serum [7,8]. Based on the evidence suggesting a higher accumulation of metformin in the liver as well as another report by Rena et al. [9], the liver is a potential target organ of metformin [10,11,12]. Several studies have suggested that metformin suppresses the hepatic gluconeogenesis resulting from glucose tolerance modulation mediated by the adenosine monophosphate-activated protein kinase (AMPK) activity [3,13,14]. Recent evidence from three studies suggests that the gut is a major target of metformin action and not the liver. First, metformin when administered intravenously, instead of orally, demonstrated no glucose-lowering effects [15,16,17]. Further, the jejunum tissue was found to exhibit a metformin concentration of up to 2000 μmol/kg of tissue, which was 30–300 times higher than the plasma concentrations [18,19,20]. The jejunum biopsy under pre-dose and post-dose of metformin demonstrated the gastrointestinal tract as a prominent target of metformin [18]. Second, the organic cation transporter (OCT) 1, expressed in the membrane of enterocytes, might be possibly involved in the absorption of metformin from the intestinal lumen [10,21]. According to Dujic et al. [22], a reduced function of OCT1 might increase the intestinal metformin concentration and the risk of gastrointestinal intolerance in the metformin-treated patients. Finally, the gut-restricted glucose-lowering effect of metformin was observed for intermediate-release metformin, extended-release metformin, and delayed-release metformin, and the same dose of metformin was more effective through those dosage forms than extended-release form [23]. Although various putative mechanisms of glucose homeostasis modulation in the gut by metformin have been proposed, more studies are needed to establish these hypotheses.

Microbiome in the human body assist in the expansion of host genomes, by facilitating the host’s metabolism and physiology [24,25]. Over the last few decades, the development of sequencing technologies and drastic progress in population-scale studies have revealed the host and microbiome relationship. Large-scale research projects on the microbiome have been actively conducted, such as the Human Microbiome Project (HMP) consortium funded by the United States National Institutes of Health (NIH) and the Metagenomics of the Human Intestinal Tract (MetaHIT) consortium funded by the European Commission [24]. A microbiome study demonstrated that the human gut microbiome abundance correlates with metabolic markers, such as adiposity, insulin resistance, and dyslipidemia [26]. Furthermore, gut dysbiosis has also been observed in T2DM patients [27,28,29,30,31,32,33,34,35]. Based on the hypothesis that metformin targets the human gastrointestinal tract, the gut microbiome has attracted attention as a key factor in the treatment of T2DM [36,37,38,39,40,41]. Thus, this review focused on the various studies related to the gut microbiome and its association with the anti-diabetic effects of metformin.

## 2. Gut Microbiome and T2DM

Over the past decade, several studies have demonstrated that patients with T2DM, obesity, or inflammatory bowel diseases often show dysbiosis in the gut microbiota [42,43,44,45]. The report by Larsen et al. [30] differentiated the composition of the gut microbiota in the T2DM patients from that in the non-diabetic adults (Table 1), and other studies have demonstrated dysbiosis in T2DM patients under different conditions, such as subject’s race and co-administration with other drugs. According to Larsen et al. [30], at the phylum level, the abundance of Firmicutes in T2DM patients was lower than that in the control group, and Bacteroidetes and Proteobacteria were more abundant than in the control group. The tendency of abundance at the phylum level was similar among the other clinical trials [29,30,33,34,35]. Furthermore, at the genus level, *Roseburia*, a butyrate-producing bacterium, was less abundant in the T2DM patients [27,29,30,32]. These results were in line with the other studies showing an increase in the abundance of *Roseburia* and insulin sensitivity after intestinal microbiota transplantation from lean donors to recipients with metabolic syndrome [46]. In addition, the abundance of *Lactobacillus* spp. was higher in T2DM patients than in the control groups [28,29,30]. The abundance of *Lactobacillus* spp. was positively correlated with blood glucose levels in the two clinical trials [29,30] and these results were consistent with those evident in a mice study [47]. The positive correlation between *Lactobacillus* spp. and the glucose levels might be due to the immunomodulatory role of *Lactobacillus* spp. [48]. Similarly, dysbiosis in T2DM patients might be due to the interaction of the gut microbiota with the host immune system, which was supported by several animal studies. In particular, the gut microbiota, which communicates with the host through pattern recognition receptors, such as toll-like receptors (TLRs), contributes to the development of insulin resistance with increased plasma LPS concentration [49,50]. According to Larsen et al. [30], the abundance of Gram-negative bacteria, which can stimulate the immune system like TLRs, was increased in T2DM patients. The role of TLRs in insulin resistance has been established through various studies. The TLR-5 deficient mice became obese and exhibited a metabolic syndrome. Further, when the gut microbiome from the TLR-5 deficient mice was transplanted to the germ-free mice, the germ-free mice showed a similar phenomenon as the TLR-5 mice [51]. In addition, Song et al. [52] reported that TLR-4 activation is associated with insulin resistance in adipocytes. Previously cited clinical studies have identified SCFA-producing bacteria as the key for dysbiosis in T2DM patients in response to the immune responses [27,28,29,32,33,34]. The gut microbiota has been considered as one of the factors affecting T2DM; thus, the gut microbiota might be considered a potential target for the treatment of T2DM. Several studies have demonstrated the positive effects of probiotics for the treatment of T2DM, such as the decrease in systemic LPS levels and improvement in insulin resistance [53,54].

## 3. Potential Mechanisms of Metformin on the Gut Microbiome

### 3.1. Regulation of Glucose Homeostasis

**Figure 2:**
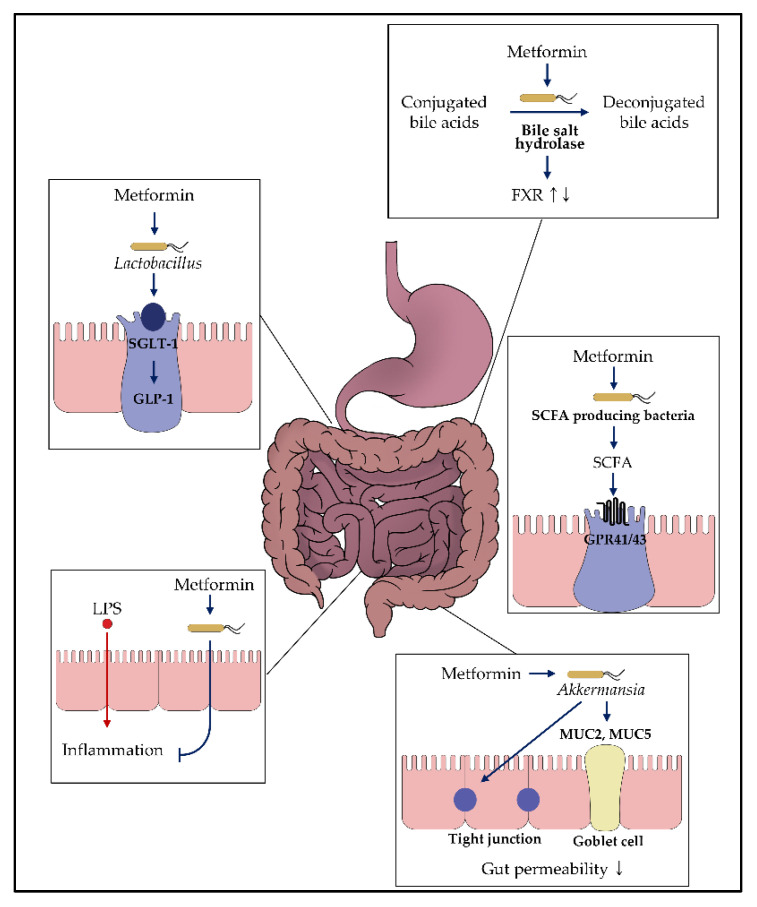
Impact of metformin on the gut microbiota. Various in vitro and in vivo studies demonstrated that metformin might exhibit glucose-modulating effects by interacting with the gut microbiome. Each box presents the putative mechanism suggested in this review. For more details, refer to the main text.

Based on the knowledge regarding metformin’s gut-restricted glucose-lowering effects, further investigations have been undertaken to understand the role of metformin in the gut (Figure 2) [23]. In particular, the upper small intestine is responsible for triggering gut peptide-dependent negative feedback signals, followed by nutrient intake [62,63]. One of the signals is glucagon-like peptide-1 (GLP-1) release via sodium-glucose cotransporter-1 (SGLT-1), which plays a dominant role in GLP-1 secretion via the transport of 3-*O*-methyl glucose [64,65,66,67,68]. In this regard, metformin exhibited an increase in GLP-1 secretion and SGLT1 expression in the upper small intestine, suggesting that metformin might interact with the upper small intestinal SGLT-1 mediated glucose-sensing pathway [69,70,71,72]. According to this hypothesis, the germ-free mice, physiologically used for the “microbial knockout” model, showed alterations in the glucose metabolism-related genes in the gut when the microbiota was inoculated from the healthy mice [73]. In addition, prebiotics and probiotics changed the gut microbiome in relation to changes in GLP-1 secretion [53,74,75]. Based on these relationships, Bauer et al. [76] demonstrated that metformin altered the upper small intestinal microbiota, resulting in the upregulation of SGLT-1 expression. Additionally, a high-fat diet in rodents reduced SGLT-1 expression, which was recovered on metformin administration [76]. This effect might be due to the alteration in microbiota in the upper small intestine, demonstrated by the transplantation of microbiota in the metformin-treated high-fat diet (HFD)-fed rats to untreated HFD-fed rats. In particular, the abundance of *Lactobacillus* exhibited significant recovery from dysbiosis, suggesting that *Lactobacillus* is related to SGLT-1 modification after metformin administration. This result was also observed in the metformin-treated HFD-fed mice with increased Sglt1 mRNA levels in the upper small intestine [59]. In addition, a previous study revealed that upregulation of SGLT-1 mediated metabolites produced by *Lactobacillus* resulted in the increased glucose uptake in Caco-2 cells, and this study supported that *Lactobacillus* might be related to the glucose modulation of metformin [77]. In terms of modulating the glucose-sensing pathway, *Lactobacillus* was shown to modulate the glucose-sensing machinery related to other pathways, not only for SGLT-1. When Caco-2 cells were incubated with the supernatant from the cultured *Lactobacillus*, there was an increase in the expression of the GPR120 gene, known to affect the expression of GLP-1 [78,79]. Furthermore, *L. gasseri*, one of the species in the genus *Lactobacillus*, was shown to affect the long-chain acyl-CoA synthetase (ACSL)-dependent glucoregulatory fatty acid-sensing pathway [80]. Thus, this evidence suggests that *Lactobacillus* plays a role in modulating glucose metabolism and might be associated with the improvement of glucose parameters in rodents and humans treated with probiotic supplements containing *Lactobacillus* [74,75].

In conclusion, metformin recovered dysbiosis in HFD-rats, and the genus *Lactobacillus* was identified as key for modulating the glucose-sensing pathway [76]. However, the mechanism by which metformin alters the abundance of *Lactobacillus* remains unknown. Thus, future studies might be required to elucidate the mechanism by which metformin affects the abundance of the gut microbiota. Furthermore, alteration of *Lactobacillus* by metformin and T2DM was not consistent between the animal and human studies, as shown in Table 1 and Table 2. For these results, Sato et al. [28] suggested that in human studies, the innate bacteria and bacteria originating from foods such as yogurt were not distinguished. In addition, Bauer et al. [76] investigated the anti-diabetic effect of metformin on the upper small intestine, comparing changes in the gut microbiome in the upper and distal intestines. Hence, these confounding factors were also regulated to unveil the relationship between metformin and the genus *Lactobacillus*.

### 3.2. Effects on Bacteria Producing Short-Chain Fatty Acid

Short-chain fatty acids (SCFAs), including acetate, propionate, butyrate, and lactate, are the major products of fermentation of undigestible food by the anaerobic bacteria. Based on the increasing number of studies on the relationship between the gut microbiota and metabolic disease, the effects of SCFAs produced by the gut microbiota on metabolic disease have attracted interest [81]. Indeed, SCFAs exhibit beneficial effects on glucose metabolism via multiple pathways, including activation of gut hormone receptors (e.g., Ffar2 and Ffar3) [81,82,83,84,85]. In particular, SCFAs can bind to the G protein-coupled receptor (GPR)-41 (referred to as FFAR3) and GPR-43 (referred to as FFAR2), expressed on enteroendocrine L cells, stimulating the release of GLP-1 and peptide YY that regulate glucose metabolism and insulin secretion [86,87]. 

Some studies have suggested that gut dysbiosis in T2DM alters the SCFA concentration. First, rodents have been used to reveal the relationship between metformin’s positive effects and SCFAs [88,89,90,91,92,93,94], specifically in the phylum Bacteroidetes, abounding in the intestine, which mainly produces acetate and propionate, imparting protective effects against insulin resistance [81,89,95,96]. The abundance of *Bacteroides*, one of the genera in the phylum Bacteroidetes, were observed to increase with metformin treatment in high-fat diet mice (Table 2) [88,89,90,91]. Following an increase in the abundance of *Bacteroides*, the concentration of SCFAs in feces of those treated with metformin was higher than that in *db/db* mice [91]. In vivo experiments using rodents, in vitro gut microbiome culture [97], and in silico modeling demonstrated similar results [98]. However, Brandt et al. [99] showed a negligible difference in the abundance of *Bacteroides*, as shown in Table 2 [89,90]. These studies used the same animal model C57BL/6J mice, but the gut microbiome was altered owing to the difference in sex, similar to the previous studies that revealed sex-dependent alterations in the gut microbiome [100,101]. *Bacteroides* were observed to be more abundant in female mice than in male mice. In this respect, Lee et al. [88] suggested that gut microbiota could be affected by hormone levels, subsequently influencing glucose and lipid metabolism [102,103] and one of the studies demonstrated that progesterone promotes the growth of oral *Bacteroides* species [104]. Although various studies have demonstrated a positive relationship between the abundance of *Bacteroides* and therapeutic effect of metformin, future studies should consider sexual effects to understand the effect of the hormones on *Bacteroides*. 

*Butyricimonas* spp., one of the genera in the phylum Bacteroidetes, produces butyrate, a moiety known to increase insulin sensitivity [105] and regulate the gut hormones [106]. *Butyricimonas* spp. were increasingly abundant in metformin-treated mice [89,91]. Besides this, the abundance of genus *Allobaculum*, a butyrate producer [107], and *Parabacteroides,* producer of succinate, [108] were also increased in metformin-treated mice [89,90,109,110]. Abundant microbiota-producing SCFAs were also observed in the human fecal samples, details regarding the same are given in Section 4.

In summary, an increase in the abundance of gut microbiota-producing SCFAs might be considered as an anti-diabetic mechanism mediated by metformin treatment. Although gut microbiota producing SCFAs (e.g., the genus *Allobaculum*, *Bacteroides*, and *Parabacteroides*) might impart beneficial metabolic homeostasis in the host, the mechanism by which metformin affects the gut microbiota is unclear. 

### 3.3. Enhancement of the Gut Permeability

Several studies have revealed that metabolic disorders are associated with increased gut permeability, which further increases the intestinal LPS permeability and induces chronic inflammation that causes insulin resistance [49,111,112,113]. The mucus layer plays an important role in maintaining gut permeability and gastrointestinal functions by providing substrates for bacterial growth adhesion and protection [114,115,116]. From this perspective, several studies suggest that colonization of several gut microbiota on the mucus layer induces diabetes or metabolic disorders from a dysbiosis-mediated high-fat diet [110,117,118].

*Akkermansia muciniphila*, belonging to the phylum Verrucomicrobia, colonizes the mucus layer of the human gastrointestinal tract and exhibits 3%–5% more microbial community in the healthy subjects than in the diabetic subjects (patients or animals) ([116,119] and references therein). *A. muciniphila* is an intestinal mucin-degrading bacterium that simultaneously stimulates mucin production, playing a key role in regulating glucose homeostasis in *A. muciniphila* [88,110,120]. Several studies have revealed that metformin treatment increases the abundance of *A. muciniphila* in the gut [88,89,90,91,99,109,110,121,122,123]. According to the study of Shin et al. [110], *A. muiciniphila* administered to HFD-fed mice showed improvement in glucose tolerance, consistent with metformin treatment in HFD-fed mice. In addition, they revealed that the proportion of *A. muciniphila* increased in metformin-treated HFD-mice and showed a positive correlation with the number of goblet cells producing mucin. As previously reported, an increase in the mucus layer by goblet cells might function as a barrier for LPS [49,111,112]. In this regard, Ahmadi et al. [92] suggested that metformin suppresses Wnt signaling, a critical pathway to regulate iSCs differentiation to goblet cells. In addition, these alterations were observed when the fecal microbiome was transplanted from the metformin-treated mice to control mice, suggesting that modulation of the gut microbiome by metformin is also associated with an increase in the goblet cells [92]. In this context, the expression of *MUC2* and *MUC5* genes, which contribute to the mucin levels, was increased in the metformin -treated HFD female mice [88]. With an increase in the expression of MUC2, several studies demonstrated that the tight-junction proteins, such as Zonulin-1 and occludin, were recovered after metformin treatment [92,94,99,124], and the intestinal permeability was reduced [94]. 

**Table 2:** Alteration of the gut microbiota-mediated metformin treatment in animal studies. ↑ (increase), ↓ (decrease), – (no alteration), NA (not applicable), Ref * (reference number).

Ref *	Animal	Study Design	Gut Microbiota	Biochemical Alterations
[76]	Rats	Metformin treatment in high-fat diet	versus without metformin treatment in high fat diet Family: *Lactobacillaceae* ↑ Genus: *Achromobacter* –, *Acinetobacter* –, *Azorhiziphilus* –, *Enterococcus* –, *Escherichia* –, *Klebsiella* –, *tobacillus* ↑, *Sarcina* –, *Stenotrophomnas* –	NA
[88]	Mice	Metformin treatment in high-fat diet	versus without metformin treatment in high fat diet α-diversity (Shannon): ↓ Phylum: Bacteroidetes ↑, Verrucomicrobia ↑ Family: *Bacteroidaceae* ↑, *Clostridiales familyXIII* ↑, *Incertae sedis* ↑, *Rikenellaceae* ↑, *Ruminococcaceae* ↑, *Verrucomicrobioaceae* ↑ Species: *Akkermansia muciniphila* ↑, *Clostridium cocleatum* ↑	Inflammation scores –
		Metformin treatment in normal diet	versus without metformin treatment in normal diet α-diversity (Shannon): – Phylum: Bacteroidetes – Family: *Rikenellaceae* ↑, *Ruminococcaceae* ↑, *Verrucomicrobioaceae* ↑ Genus: *Alistipes* spp. ↑, *Akkermansia* spp. ↑, *Clostridium* spp. ↑	NA
[89]	Mice	Metformin treatment for 16 weeks in high-fat diet	versus without metformin treatment in high fat diet α-diversity (observed OTU): – Phylum: Bacteroidetes ↑, Firmicutes ↓, Verrucomicrobia ↑ Genus: *Akkermansia* ↑, *Bacteroides* ↑, *Butyricimonas* ↑, *Parabacteroides* ↑	IL-6 mRNA ↓ IL-1β mRNA ↓
[90]	Mice	Metformin treatment for 24 weeks in high-fat diet	versus without metformin treatment in high fat diet α-diversity (Shannon): ↓ Phylum: Bacteroidetes ↑, Firmicutes ↓, Verrucomicrobia ↑ Family: *Desulfovibrionaceae* Genus: *Akkermansia* ↑, *Bacteroides* ↑, *Christensenella* ↑, *Coprococcus*↓, *Dorea* ↓, *Lachnoclostridium* ↓, *Parabacteroides* ↑, *Papillibacter* ↓, *Oscillospira* ↓, *Ruminococcus* ↓, *Desulfovibrio* ↓, *Muribaculum* ↓	Plasma threonine ↓, methionine sulfoxide ↓, Tetradecanoylcarnitine ↓, Hexadecenoylcarnitine ↓
[91]	Mice	Metformin treatment in obese mice (*db*/*db* mice)	versus without metformin treatment in obese mice (*db*/*db* mice) α-diversity (Shannon): ↑ Genus: *Akkermansia* ↑, *Butyricimonas* ↑, *Clostridium* ↓, *Coprococcus* ↑, *Dehalobacterium* ↑, *Dorea* ↑, *Lactobacillus* ↑, *Oscillospira* ↑, *Parabacteroides* ↓, *Paraprevotella* ↑, *Prevotella* ↓, *Proteus* ↓, *Ruminococcus* ↑	Total SCFA concentration in feces ↑ Acetic acid ↑, Butyric acid ↑ LPS levels ↓
[92]	Mice	Metformin treatment in high-fat diet	versus without metformin treatment in high fat diet α-diversity (Shannon, evenness): – Phylum: Bacteroidetes ↑ Family: *Coriobacteriaceae* ↓, *Ruminococcaceae* ↑, *S24_7* ↑, *Veilonellaceae* ↓ Genus: *Dorea* ↓, *Dehalobacterium* ↓, *Lactobacillus* ↓, *Lactococcus* ↑, *Roseburia* ↓, *SMB53* ↓	IL-6 ↓, IL-1β ↓, TNF α ↓ Taurine ↑, Butyrate ↑, Total Bile acids ↑, Propionate ↑, Leucine ↑, Creatinine ↓, Sarcosine ↓, Glutamate ↓, Pyruvate ↓, Formate ↓
[93]	Rats	Metformin treatment in high-fat diet combined with a low dose streptozocin	versus without metformin treatment in high fat diet α-diversity (Simpson, Shannon): ↑ Class: Coriobacteriia ↑ Family: *S24_7* ↑	Total SCFAs ↑, Butyric acid ↑, Isovaleric acid ↑
[94]	Rats	Metformin treatment in high-fat diet combined with a low dose streptozocin	versus without metformin treatment in high fat diet α-diversity (Chao1): ↑ Family: *S24_7* ↓ Genus: *Anaerotruncus* ↑, *Escherichia-Shiegella* ↓, *Eubacterium* *xylanophilum* ↑, *Lachnospiraceae NK4A136* ↑, *Lachnospiraceae-UCG_006* ↑, *Roseburia* ↑	Serum LPS ↓, Serum CRP↓, Serum TNF α ↓, Serum IL-6 ↓ Propionate in cecum ↑, Butyrate in cecum ↑
[99]	Mice (female)	Metformin treatment in fat, fructose and cholesterol rich diet	versus without metformin treatment in fructose and cholesterol rich diet Family: *Alloprevotella* ↓ Genus: *Bacteroides* –, *Romboutsia* ↓ Species: *Akkermansia muciniphila* –, *Lactobacillus animalis* ↓	TNF α ↓ Endotoxin ↓
[109]	Mice	Metformin treatment for 5 weeks in high-fat diet combined with a low dose streptozocin	versus without metformin treatment in high fat diet α-diversity (observed OTU): – Genus: *Akkermansia* ↑, *Bacteroides* spp. ↓	NA
[110]	Mice	Metformin treatment in high-fat diet	versus without metformin treatment in high fat diet Phylum: Verrucomicrobia ↑, Genus: *Akkermansia* ↑, *Alistipes* ↑, *Anaerotruncus* ↓, *Blautia* ↓, *Lactococcus* ↓, *Lactonifactor* ↓, *Lawsonia* ↓, *Odoribacter* ↓, *Parabacteroides* ↓	IL-6 mRNA ↓ IL-1β mRNA ↓
[121]	Mice	Metformin treatment for 30 days	versus without metformin treatment in healthy mice α-diversity (Shannon): – Class: Lachnopiraceae ↓, Porphyromonadaceae ↑, Prevoltellaceae ↑, Rhodobacteraceae ↓, Rikenellaceae ↑, Verrucomicrobiaceae ↑	NA
[123]	Rats	Metformin treatment in high-fat diet	versus without metformin treatment in high fat diet α-diversity (Shannon): ↓ Phylum: Bacteroidetes –, Firmicutes –, Proteobacteria ↑ Species: *Akkermansia* ↑, *Allobaculum* ↑, *Bacteroides* ↑, *Blautia* ↑, *Butyricoccus* ↑, *Clostridium* ↓, *Klebsiella* ↑, *Lactobacillus* ↑, *Parasutterella* ↑, *Phascolarctobacterium* ↑, *Prevotella* ↑, *Roseburia* ↓	NA
[124]	Rats	Metformin treatment in high-fat diet combined with a low dose streptozocin	versus without metformin treatment in high fat diet α-diversity (Chao1, Shannon): ↑ Phylum: Bacteroidetes ↑, Firmicutes ↓, Proteobacteria ↓ Order: Clostridiales ↑, Enterobacteriales ↓, Lactobacillales ↑ Genus: *Akkermansia* ↑, *Desulfovibrio* ↓, *Lachnospiraceae NK4A136* ↓, *Lactobacillus* ↑, *Roseburia* ↑	NA
[125]	Mice	Metformin treatment for 3 weeks in high-fat diet	versus without metformin treatment in high fat diet α-diversity (Shannon, evenness): – Genus: *Akkermansia* ↑, *Allobaculum* ↓, *Clostridium* ↓, *Enterococcus* ↓, *Lactococcus* ↓, *Leuconostoc* ↓, *Oscillospira* ↑, *Parabacteroides* ↑, *Prevotella* ↑, *Ruminococcus* ↓, *Streptococcus* ↓	NA
[126]	Mice with	Metformin treatment for 5 weeks in high-fat diet combined with a low dose streptozocin	versus without metformin treatment in high fat diet α-diversity (Chao1): ↓ Phylum: Bacteroidetes ↓, Firmicutes ↑, Proteobacteria ↓ Genus: *Lactobacillus* ↑	NA
[127]	Mice	Metformin treatment in high-fat diet	versus without metformin treatment in high fat diet α-diversity (Shannon, evenness): – Species: *Bacteriodetes fragilis* ↓, *Escherichia coli* ↓	Serum endotoxin ↓ IL-6 ↓, TLR4 ↓
[128]	Rats	Metformin treatment in high-fat diet combined with a low dose streptozocin	versus without metformin treatment in high fat diet α-diversity (Chao1): ↑ Phylum: Bacteroidetes ↑, Proteobacteria ↓, Verrucomicrobia ↓ Family: *Alcaligenaceae* ↑, *Peptococcaceae* ↑, *Prevotellaceae* ↑, *S24_7* ↑ Genus: *Prevotella* ↑, *Sutterella* ↑, *02d06* ↑, *rc4* ↑	IL-6 mRNA in pancrease ↓, TNF α mRNA in pancrease ↓, LPS ↓
[129]	Rats	Metformin treatment in high-fat diet combined with a low dose streptozocin	versus without metformin treatment in high fat diet Genus: *Bifidobacterium* ↑, *Lactobacillus* ↑ Species: *Clostridium perfringens* ↓, *Escherichia coli* ↓	Plasma endotoxin ↓, Total SCFAs in cecum ↑, Lactic acid in cecum ↑, Acetic acid in cecum ↑
[130]	Rats	Metformin treatment in Otsuka Long-Evans Tokushima Fatty (OLETF) rats	versus without metformin treatment Genus: *Akkermansia* ↑, *Prevotella* ↓, *Roseburia* ↑ Species: *Escherichia coli* ↓	Serum endotoxin ↓, Fecal endotoxin ↓, serum TNF α ↓, serum IL-6 ↓
[131]	Rats	Metformin treatment in Zucker diabetic fatty rats	versus without metformin treatment α-diversity (Shannon): – Phylum: Bacteroidetes –, Firmicutes –↑, Proteobacteria ↓, Tenericutes –, Verrucomicrobia ↑ Genus: *Lactobacillus* ↑ Species: *Lactobacillus intestinalis* ↑, *Lactobacillus johnsonii* ↑	NA

Furthermore, *A. muciniphila* has been shown to strengthen the intestinal barrier by increasing the expression of the tight junction proteins [132,133,134]. Based on this supporting evidence, metformin might be capable of reducing gut permeability via increased expression of the mucin and tight-junction proteins. However, Shin et al. [110] revealed that there was no substantial change in the gut permeability of LPS and suggested that increased goblet cells may act as a barrier against LPS by producing immune-effector molecules [135,136]. These differences among studies might be due to the duration of treatment, dosage, or the amount of *A. muciniphila* in the gut. In addition, the in vitro culture [132,133,134] using bacteria is complex; thus, it might be different from the in vivo studies [88,89,90,91,99,109,110,121,122,123].

In conclusion, the increase in the abundance of *A. muciniphila* by metformin treatment promotes mucin production, which might recover the increased gut permeability induced by high-fat diets or metabolic disorders. However, until now, the direct effects of metformin on cellular pathways to increase *A. muciniphila* remain unclear. Thus, further investigation to identify the physiological pathway by which metformin increases *A. muciniphila* will help understand the unidentified effects of metformin on the gut microbiota. 

### 3.4. Modulation of the Immune Response

In the past decade, accumulating evidence from a variety of animal models or clinical studies has shown that metabolic disorders, including T2DM, are associated with chronic or subacute tissue inflammation in the adipose tissue and liver, causing insulin resistance [137,138,139,140,141]. Several studies in T2DM, have reported that metformin modulates inflammation via inflammatory modulating signaling pathways, such as STAT3 signaling [142] or the NF-κB (nuclear factor kappa light chain enhancer of activated B cells) signaling pathway [94,143,144]. Metformin directly suppresses the release of an inflammatory cytokine such as interleukin 6 (IL-6), interleukin 1β (IL-1β), and TNF-α (tumor necrosis factor– α) [92,127,128]. Thus, several studies have reported alterations in the expression of IL-6 following metformin treatment, and some reports have even showed that the alteration of microbiota due to metformin treatment was related to the modulation of inflammation. 

In detail, *A. muciniphila*, the abundance of which increased upon metformin treatment as mentioned above, also exhibited anti-inflammatory effects in the gut, consistent with previous studies that revealed the anti-inflammatory effect of *A*. *muciniphila* [110,145,146]. Shin et al. [110] demonstrated that decreased regulatory T cells, a regulator of immune responses, in the stromal vascular fraction of the HFD-control mice was recovered by *A. muciniphila* and metformin treatment. Furthermore, the *IL-6* and *IL-1β* mRNA levels were significantly decreased in *A. muciniphila* on metformin treatment [110]. In this context, a negative correlation can be drawn between the abundance of *A. muciniphila* upon metformin treatment and inflammatory markers, such as inflammatory cytokines or LPS concentration [89,91,121,122]. These effects of *A. muciniphila* on inflammation have also been demonstrated in human studies (Clinical Trials.gov Identifier: NCT02637115) with fewer inflammation markers and improved insulin sensitivity [147]. 

Likewise, the abundance of *Bacteroides* and *Butyricimonas* also increased upon metformin treatment [89,90,91,97,98,109,123]. In particular, Lee et al. [89] revealed that IL-6 expression negatively correlated with the abundance of *Bacteroides* and *Butyricimonas*. Above all, IL-6 possesses not only pro-inflammatory effects but also attenuates insulin signaling in adipocytes [148,149,150]. Thus, decreased IL-6 expression on metformin treatment may contribute to its anti-diabetic effect. In addition, Lee et al. [89] showed that the expression of IL-1β, which is related to insulin resistance, decreased while the abundance of *Bacteroides* and *Butyricimonas* was increased. The tendency to decrease the expression of IL-6, IL-1β and TNF-α was also observed in other studies, but the types of bacteria that correlated with the expression of inflammatory cytokines were different [92,94,128,130]. With the inhibition of pro-inflammatory cytokines, modulation of the inflammatory signaling pathway is a potential mechanism to attenuate inflammation. The TLR/NF-κB signaling pathway also plays a role in intestinal inflammation [151]. Zhang et al. [91] demonstrated that the metformin-treated group exhibited downregulation of the intestinal TLR/NF-κB signaling activities. A similar result was observed wherein phosphorylation of IKKα/β upstream of NF-κB signaling was decreased in metformin-treated mice [99]. In addition, abundance of other gut bacteria increased in the metformin-treated group and were known to interact with the host immune response. For example, *Roseburia* is more abundant in the metformin-treated group and is known to inhibit the activity of NF-κB [92,94,124,130,152,153]. In addition, the genus *Lactobacillus* and several *Lactobacillus* species have been shown to modulate inflammation, as reported in previous studies [154,155,156,157]. Thus, future studies should be warranted to unveil how metformin prevents the host inflammatory response related to the alteration of gut bacteria.

To conclude, various inflammatory markers were correlated with the alteration of bacteria on metformin treatment. Furthermore, these effects have also been supported by other studies that demonstrated the therapeutic effects of metformin on inflammatory diseases (e.g., non-alcoholic fatty liver disease and polycystic ovary syndrome) through interaction with the gut microbiota [99,158].

### 3.5. Actions on the Circulation of the Bile Acids

Bile acids are synthesized from cholesterol in the liver and secreted into the intestine, following which cholic acid and chenodeoxycholic acid are converted to secondary bile acids, such as deoxycholic acid and lithocholic acid, via enzymes and gut microbiota. For several decades, bile acids have been shown to play a role in glucose, lipid, and energy metabolism [159]. The modulation effects of bile acids on several metabolic pathways are mainly via binding to several intracellular nuclear receptors, including farnesoid X receptor (FXR), pregnane X receptor (PXR), and cell surface G protein-coupled receptors (GPCRs) ([160] and references therein). In this regard, metformin showed an inhibitory effect on the bile acid resorption, resulting in increased exposure of the gut to bile acids [92,161,162,163]. Napolitano et al. [55] demonstrated the effect of metformin on bile acid in a clinical trial in T2DM patients. Extended exposure to bile acid might allow bile acids to bind to the intestinal FXR. Thus, the glucose-modulating effect of metformin via bile acids seems to be related to the FXR signaling. However, the glucose-modulating effect mediated by FXR remains controversial. There is some evidence that inactivation of FXR results in better glucose control and increased GLP-1 secretion [164,165,166]. For example, FXR-deficient mice exhibit increased GLP-1 expression and improved glucose metabolism [164]. In contrast to these results, some studies suggested that activation of FXR via FXR agonists improves glucose tolerance and insulin sensitivity [167,168,169,170,171]. Thus, the glucose-modulating effect of metformin via bile acid circulation has not yet been clarified. Recently, a study revealed that metformin acts on the *B. fragilis-glycoursodeoxycholic* acid (GUDCA)-intestinal FXR axis, improving hyperglycemia [59]. GUDCA, a conjugated bile acid, is deconjugated by the gut microbiome and is demonstrated to be an FXR antagonist. Sun et al. [59] revealed that metformin inhibited the deconjugation of GUDCA through the activity of the bile salt hydrolase of *B. fragilis*, resulting in an increased GUDCA concentration. This result is consistent with the correlation between GUDCA levels in stool and the presence of *B. fragilis*. Additionally, the abundance of *Lactobacillus sanfrancisensis*, contained in the genus *Lactobacillus* known to affect intestinal FXR signaling, was increased in the metformin-treated HFD-fed mice [80].

Taken together, metformin has a role in modulating glucose homeostasis via the regulation of the bile acid circulation. Conflict in the function of FXR in glucose homeostasis might be due to different agonists and antagonists for FXR (e.g., intestinal FXR agonist or whole-body FXR agonist) [160]. Furthermore, bile acid pools in mice and humans are known to be quite different and might have a conflicting role in FXR. As a result, further studies could be conducted by considering these confounding factors.

## 4. Relationships between Metformin and Gut Microbiome in Human Studies

The glucose-modulating effect of metformin on the gut microbiome has been evaluated in various clinical trials. The first clinical study that observed the relationship between metformin and the gut microbiome was conducted as an open-label, single-group study in T2DM patients [55]. In this study, they demonstrated alterations in the composition of the gut microbiome, glucose hormone, glucose-related parameters, and bile acid concentration in feces. A similar tendency was observed in the present study, despite minor differences in the gut microbiome composition (Table 1).

First, at the phylum level, the alterations in the abundance of Firmicutes and Bacteroidetes were remarkable on comparing visits 3 (non-treatment) and 4 (metformin treatment). Although there were differences among subjects, the abundance of Firmicutes was commonly increased, whereas that of Bacteroidetes was decreased after metformin treatment. This result is in line with the previous finding that the Firmicutes/Bacteroidetes ratios were considered a predictor for metabolic disease such as T2DM or obesity in several human studies [30,172,173]. The Firmicutes/Bacteroidetes ratios were decreased in the T2DM patients, and this phenomenon was recovered by metformin treatment in several clinical studies [55,56]. In contrast to these results, some studies did not show an alteration in the ratio of Firmicutes to Bacteroidetes [60]. Inconsistencies among studies could be considered for the following reasons. The phyla Firmicutes and Bacteroidetes are the most abundant bacteria in the human gut and include a large number of bacterial species. Thus, a comparison of the Firmicutes and Bacteroidetes ratio is considered too simple to evaluate metabolic disease or improvement. In addition, the difference might be attributed to the compositional difference between the stool and biopsy specimens. Clinical studies in this review used fecal samples to analyze the gut microbiome, but previous studies have shown differences in the microbial compositions of biopsy and fecal samples [42,174]. In particular, the mucosa-associated microbiota, to which the phylum Firmicutes is enriched, exhibited compositional differences in biopsies derived from colon and stool samples [175,176]. Thus, Hollister et al. [174] suggested biopsy or surgical specimens for the evaluation of mucosa-associated microbiota. For these reasons, further investigations require the validity of the Firmicutes/Bacteroidetes ratio as a relevant marker for metabolic diseases. At the genus level, it is noteworthy that *Escherichia,* including *Escherichia/Shigella* and *Escherichia coli*, exhibited a significant increase in T2DM patients upon metformin treatment. An increase in the abundance of *Escherichia/Shigella* upon metformin treatment was also observed in the other clinical trials including healthy volunteers [29,31,56,60,61]. Forslund et al. [31] suggested that metformin administration creates a competitive environment for *Escherichia* coli using nitrate or other energy sources, resulting in changes in the abundance of the gut microbiome [177]. Wu et al. [56] also demonstrated a change in the abundance of *E. coli* as an indirect effect of metformin treatment in the in vitro gut simulation. Elbere et al. [60] demonstrated that the abundance of *Escherichia*/*Shigella* before metformin treatment is associated with side effects. In this study, the increased presence of *Escherichia/Shigella* showed mild and severe side effects; however, this level was lower than the detection limit in the no-side-effect group. Thus, increased abundance of *Escherichia* was considered as a marker for the gastrointestinal side effects of metformin. Forslund et al. [31] suggested that side effects derived from *Escherichia* are due to an increase in lipopolysaccharide synthesis or sulfate metabolism potential, known to contribute to intestinal bloating [31,178,179,180,181].

In contrast, the abundance of *Intestinibacter* spp. decreased in T2DM patients treated with metformin in several clinical studies [31,56,61]. Until now, the role of *Intestinibacter* is still unclear, Forslund et al. [31] suggested that *Intestinibacter* showed resistance to oxidative stress and degradation of fucose, indicating indirect mucus degradation through analysis of SEED (http://pubseed.theseed.org/, accessed on 30 March 2021) [182] and gut microbial modules (GMM) functional annotations.

In addition, *A. muciniphila*, which is positively correlated with metformin treatment, showed a less clear link in human studies. Although Wu et al. [56] demonstrated an increase in *A. muciniphila* in the in vitro pure cultures, there was no correlation between the abundance of *A. muciniphila* and % hemoglobin A1c. Furthermore, clinical studies in healthy volunteers showed no change in the abundance of *A. muciniphila* when they were treated with metformin [60,61]. The reasons for these differences might be considered to be affected by factors dependent on individuals, such as fibers [183], polyphenol availability [184,185], immune response [186,187], and age [188,189]. Thus, it might be difficult to conclude the role of *A. muciniphila* in humans as a major contributor to the anti-diabetic effect of metformin, although improvements in the metabolic parameters were observed in the *A. muciniphila*-treated human studies.

From the perspective of biochemical alterations upon metformin treatment, there were some differences in the bile acid and SCFA concentrations in the feces. They found that metformin exposure increased the excretion of bile acid in feces, consistent with the inhibitory effect of metformin on the resorption of bile acids [161,162,163]. In addition, the abundance of Firmicutes and Bacteroidetes correlated with the bile acid concentration and gut peptide, suggesting that metformin indirectly regulates the secretion of gut hormones via bile acid metabolism. Increased SCFA concentration in feces or an increase in the abundance of SCFA-producing bacteria has been observed in human studies [31,56,57,58]. In particular, Wu et al. [56] only demonstrated that the concentration of SCFA in fecal samples, resulting in formation of butyrate and propionate, substantially increased on metformin treatment. This result is consistent with animal studies that showed that metformin increases SCFA-producing bacteria [88,89,90,91,109,110]. Thus, these clinical results support the hypothesis that metformin exerts beneficial effects via bile acids and SCFAs. 

The clinical studies discussed in this review exhibited differences among studies, including observed taxonomic groups in metformin treatment and diversity in abundance. As far as diversity is concerned, only a few subjects were engaged in the clinical study, resulting in no statistical difference in the diversity of the gut microbiome. This issue has been inconsistent in clinical trials (Table 1). Forslund et al. [31] conducted a meta-analysis of metagenomic data from Swedish, Danish, and Chinese individuals. In this study, gut microbiome was less rich T2DM patients without metformin treatment; this richness slightly recovered, almost as much as that in the control group, in T2DM patients on metformin treatment [31]. The diversity was also shown to decrease in the Chinese T2DM patients [79], which was consistent with the results from the study by Forslund et al. [31]. 

These differences might be derived from the dosage, study duration, disease state, race differences, and sample size. Thus, to elucidate the anti-diabetic effect of metformin via modulation of the gut microbiome, clinical studies in ethnic-controlled environments or comparisons among ethnicities are required. Indeed, clinical studies in various populations have been conducted at ClinicalTrial.gov (assessed on 30 March 2021) (Table 3). Most of the research to date has revealed taxonomic groups in the gut at the genus level, and not at the species level, due to technical limitations. To counter this limitation, recent studies introduced a gut microbiome analysis method to make a possible profile at the species level [190,191,192]. In the future, these methods to analyze the gut microbiome could help clarify the relationship between metformin and the gut microbiome.

## 5. Perspective and Conclusions

Metformin, the first-line medicine for T2DM, has been investigated to elucidate its antidiabetic effects. In the recent decade, with progress in metagenomic technology, the role of the gut microbiome in host metabolism has been highlighted. Several studies have demonstrated that some medicines alter the composition of the gut microbiome, and this phenomenon might be considered as one of the mechanisms for treatment. In this regard, some scientists have also investigated the relationship between metformin and the gut microbiome. In this review, we focused on the current knowledge about the glucose-modulating effect of metformin on the gut microbiome. Taken together, metformin might affect the intestinal microbiome via the modulation of inflammation, gut permeability, glucose homeostasis, and abundance of SCFA-producing bacteria. However, it is still not clear how metformin modulates glucose homeostasis via the gut microbiota. Hence, elucidation of the mechanism to treat T2DM with intestinal microbiota remains a challenge for future research. In addition, without metformin, the composition of intestinal microbiota is affected by several factors, including genetics, sex, foods, lifestyle, and other medications [193,194,195]. Thus, to fully elucidate the mechanism, these confounding factors must be taken into account to determine the sole effects of metformin on the gut microbiota. However, control of the interventions is quite difficult in clinical studies. Furthermore, fecal microbiome transplantation could be recommended to validate the mechanism of action via gut microbiome-based Koch’s postulates [89,196]. However, it is difficult to conduct fecal microbiome transplantation to validate the mechanism of action via the gut microbiome in clinical settings. In addition, most of the studies dealt with within this review conducted functional annotation, categorizing microbial functions at the community level; however, it is difficult to suggest a mechanistic explanation for how these functions arise [197,198,199]. In this context, computational methods have been used as supportive tools to help elucidate the mechanism of action, and several experiments could be supportive to validate the hypothesis. Pryor et al. [98] suggested a notable approach to identify drug-nutrient interactions, combined with the in silico microbial modeling. Additionally, the results from the suggested methodology are consistent with those of other human studies [31,56,98]. In addition, Rosario et al. [122] showed the contribution of four bacteria (*Escherichia* spp., *Akkermansia muciniphila*, *Intestinibacter bartlettii*, and *Subdoligranulum variable*) in the physiology of metformin-treated T2DM patients through genome-scale models, and this study was in line with the previous observations [200,201]. Recent studies have demonstrated the drug-microbiome-host relationship via various in silico studies [122,202,203]. Hence, combined with computational methods and experimental methods, it might be helpful to understand the mechanism of action of metformin on the gut microbiome.

Understanding how metformin modulates glucose metabolism through gut microbiota may be helpful for patients with T2DM who fail upon metformin treatment, and it is possible to inform dietary guidelines to maximize the therapeutic effects, such as using probiotic products and reducing the gastrointestinal side effects [38,98,204].

## Figures and Tables

**Table 1:** Alteration of the gut microbiota biochemical properties in the T2DM patients compared to the healthy subjects or alteration in the metformin treatment compared non-treatment T2DM patients or healthy subjects. ↑ (increase), ↓ (decrease), – (no alteration), NA (not applicable), Ref * (reference number).

Ref *	Population	Study Design	Gut Microbiota	Biochemical Alterations
[27]	Chinese	T2DM patients (*n* = 170)	versus Healthy subjects (*n* = 174) Family: *Lachnospiraceae* ↑, *Erysipelotrichaceae* **↓** Genus: *Alistipes* ↑, *Clostridium* ↑, *Eubacterium* **↓,** *Faecalibacterium* **↓,** *Subdoligranulum* ↑, *Parabacteroides* ↑ Species: *Akkermansia muciniphila* ↑, *Bacteroides intestinalis* **↑,** *Clostridium bolteae* **↑,** *Clostridium hatheway* ↑, *Clostridium ramosum* ↑, *Clostridium symbiosum* ↑, *Eggerthella lenta* ↑, *Escherichia coli* ↑, *Eubacterium rectale* **↓,** *Faecalibacterium prausnitzii* **↓,** *Haemophilus parainfluenzae* **↓,** *Roseburia intestinalis* **↓,** *Roseburia inulinivorans* **↓**	NA
[30]	Danish	T2DM patients (*n* = 18)	versus Healthy subjects (*n* = 18) α-diversity (Chao 1): – Phylum: Bacteroidetes ↑, Firmicutes ↓, Proteobacteria ↑ Class: Bacilli ↑, Bacteroidetes ↑, Betaproteobacteria ↑, Clostridia ↓ Genus: *Akkermansia* ↑, *Alistipes* ↑, *Bacteroides* ↓, *Bifidobacterium* ↓, *Bilophila* ↑, *Catenibacterium* ↓, *Dialister* ↑, *Dorea* ↑, *Erysipelotrichaceae IS* ↑, *Faecalibacterium* ↓, *Lachnospiraceae IS* ↓, *Lactobacillus* ↑, *Parabacteroides* ↑, *Prevotella* ↑, *Roseburia* ↓, *Ruminococcus* ↓, *Sporobacter* ↑, *Subdoligranulum* ↓, *Succinivibrio* ↑, *Sutterella* ↑ Species: *Dorea longicatena* ↓	NA
[32]	Chinese	T2DM patients (*n* = 13)	versus Healthy subjects (*n* = 44) α-diversity (Chao 1, Shannon index): ↓ Class: Clostridia ↑, Clostridiales ↑ Family: *Lachnospiraceae* ↑ Genus: *Abiotrophia* ↑, *Bacteroides* ↓, *Collinsella* ↑, *Dorea* ↑, *Eubacterium* ↑, *Haemophilus* ↓, *Megamonas* ↓, *Peptostreptococcus* ↑, *Prevotella* ↑, *Roseburia* ↓, *Ruminococcus* ↑, *Sporobacter* ↑, *Subdoligranulum* ↑	NA
[34]	Pakistani	Obese-T2DM patients (*n* = 40)	versus Healthy subjects (*n* = 20) α-diversity (Shannon index): ↓ Phylum: Bacteroidetes ↓, Elusimicrobia ↓, Firmicutes ↓, Proteobacteria ↓, Verrucomicrobioa ↓ Class: Bacilli ↓, Bacteroidia ↓, Clostridia ↑, Coriobacteriia ↑, Deltaproteobacteria ↓, Elusimicrobia ↓, Gammaproteobacteria ↓, Negativicutes ↑, Genus: *Allisonella* ↑, *Bacillus* ↓, *Christensenellaceae_R_7* ↑, *Dialister* ↑, *Escherichia_Shigella* ↓, *Eubacterium coprostanoligenes groups* ↑, *Lactobacillus* ↑, *Prevotella_9* ↓, *Ruminococcus_2* ↓, *Subdoligranulum* ↑	NA
**Metformin Treatment Effects in T2DM Patients**				
[28]	Japanese	T2DM patients (*n* = 50)	versus normal subjects (*n* = 50) Genus: *Atopobium cluster* ↓, *Lactobacillus* ↑, *Prevotella* ↓ Species: *Clostridium coccoides* ↓, *Lactobacillus plantarum* ↑, *Lactobacillus reuteri* ↑	Fecal organic acids ↓ Acetic acid ↓ Propionic acid ↓ Fecal isovaleric acid ↑ CRP ↑, IL-6 ↑
		Metformin treated T2DM (*n* = 17)	versus non treated T2DM (*n* = 33) Family: *Enterobacteriaceae* ↑ Genus: *Staphylococcus* ↑ Species: *Clostridium coccoides* ↓, *Lactobacillus plantarum* ↑, *Lactobacillus reuteri* ↑	NA
[29]	European old woman	T2DM patients (*n* = 53)	versus normal glucose tolerance (*n* = 43) Class: Clostridiales ↓ Family: *Coriobacteriaceae* ↓ Genus: *Alistipes* ↓, *Clostridium* ↓, *Roseburia* ↓, Species: *Bacteroides intestinalis* ↓, *Eubacterium eligens* ↓, *Lactobacillus gasseri* ↑, *Streptococcus mutans* ↑	C-peptide ↑
		Metformin treated T2DM (*n* = 20)	versus non treated T2DM (*n* = 33) Genus: *Clostridium* ↓, *Escherichia* ↑, *Eubacterium* ↓, *Klebsiella* ↑, *Salmonella* ↑, *Shigella* ↑ Species: *Escherichia coli* ↑	NA
[31]	Danish	T2DM patients (*n* = 75)	versus normal subjects (*n* = 277) Family: *bp Clostridiales* ↓, *Peptostreptococcaceae* ↓ Genus: *Akkermansia* ↓, *Acidaminococcus* ↑, *Bilophila* ↑, *Collinsella* ↑, *Coprococcus* ↓, *Escherichia* ↑, *Holdemania* ↑, *Lactobacillus* ↑, *Parabacteroides* ↑, *Roseburia* ↓, *Veillonella* ↓	NA
		Metformin treated T2DM (*n* = 58)	versus non treated T2DM (*n* = 17) Family: *Peptostreptococcaceae* ↓ Genus: *Akkermansia* ↑, *Bilophila* ↓, *Escherichia* ↑, *Holdemania* ↑, *Roseburia* ↑, *Veillonella* ↓	NA
	Swedish female	T2DM patients (*n* = 53)	versus normal subjects (*n* = 92) Family: *Peptostreptococcaceae* ↓ Genus: *Lactobacillus* ↑	NA
		Metformin treated T2DM (*n* = 20)	versus non treated T2DM (*n* = 33) Family: *bp Clostridiales* ↓, *Peptostreptococcaceae* ↓ Genus: *Bilophila* ↑, *Escherichia* ↑, *Holdemania* ↓, *Lactobacillus* ↑, *Roseburia* ↓, *Veillonella* ↓	NA
	Chinese	T2DM patients (*n* = 71)	versus normal subjects (*n* = 185) Family: *bp Clostridiales* ↓, *Peptostreptococcaceae* ↓, Genus: *Acidaminococcus* ↑, *Bilophila* ↑, *Collinsella* ↑, *Coprococcus* ↓, *Escherichia* ↑, *Haemophilus* ↓, *Holdemania* ↑, *Lactobacillus* ↑, *Oscillibacter* ↑, *Roseburia* ↓, *Veillonella* ↓	NA
		Metformin treated T2DM (*n* = 15)	versus non treated T2DM (*n* = 56) Family: *bp* *Clostridiales* ↑, *Peptostreptococcaceae* ↓ Genus: *Bilophila* ↑, *Collinsella* ↑, *Escherichia* ↓, *Holdemania* ↑, *Parabacteroides* ↑, *Roseburia* ↑, *Subdoligranulum* ↑, *Veillonella* ↓	NA
[33]	Chinese	T2DM patients (*n* = 26)	versus normal subjects (*n* = 50) α-diversity (Shannon index): ↓ Phylum: Firmicutes ↓ Class: Fusobacteriia ↑ Family: *Enterobacteriaceae* ↓, *Erysipelotrichaceae* ↑, *Erysipelotrichaceae* ↑, *Porphyromonadaceae* ↑ Genus: *Faecalibacterium* ↓, *Fusobacterium* ↑, *Lactobacillus* ↑, *Ruminococcus* ↓	NA
		Metformin treated T2DM (*n* = 51)	versus non treated T2DM (*n* = 26) α-diversity (Shannon index): – Phylum: Actinobacteria ↓ Family: *Enterobacteriaceae* ↓, *Spirochaetaceae* ↑, *Turicibacteraceae* ↑ Genus: *Fusobacterium* ↑, *Turicibacter* ↑	NA
[55]	British	On metformin T2DM (visit 1 and 4, *n* = 12)	versus off metformin T2DM (visit 2 and 3, *n* = 12) Genus: *SMB53* ↓, *Adlercreutzia* ↓, *Eubacterium* ↑	Serum bile acids ↓ Fecal bile acids ↑ GLP-1 ↑
[56]	Spanish	Metformin treated T2DM for 4 months (*n* = 22)	versus before metformin treatment in T2DM (*n* = 22) Phylum: Proteobacteria ↑, Firmicutes ↑ Genus: *Actinetobacter* ↑, *Alkaliphilus* ↓, *Citrobacter* ↑, *Cronobacter* ↑, *Dermcoccus* ↑, *Desulfurispirillum* ↑, *Dickeya* ↑, *Edwardsiella* ↑, *Enterobacter* ↑, *Erwinia* ↑, *Escherichia* ↑, *Holdemania* ↓, *Intestinibacter* ↓, *Klebsiella* ↓, *Methylobaciilus* ↑, *Pantoea* ↑, *Pectobacterium* ↑, *Photorhabdus* ↑, *Providencia* ↑, *Pseudomonas* ↑, *Rahnella* ↑, *Rheinheimera* ↑, *Salmonella* ↑, *Subdoligranulum* ↓, *Xanthomonas* ↑, *Xenohabdus* ↑, *Yersinia* ↑ Species: *Akkermansia muciniphila* ↑, *Bifidobacterium adolescentis* ↑	Fecal propionate, butyrate, lactate and succinate ↑ Plasma bile acids ↑
[57]	Colombian	T2DM patients (*n* = 28)	versus normal subjects (*n* = 84) Genus: *Enterococcus casseliflavus* ↓, *Clostridiaceae* 02d06 ↑, *Prevotella* ↑	NA
		Metformin treated T2DM (*n* = 14)	versus non treated T2DM (*n* = 14) Genus: *Bacnesiellaceae* ↓, *Butyrivibrio* ↑, *Clostridiaceae* 02d06 ↓, *Megasphaera* ↑, *Oscillospira* ↓, *Prevotella* ↑	NA
[58]	Scandinavian	Metformin treated T2DM (*n* = 23)	versus non treated T2DM (*n* = 7) Family: *Enterobacteriaceae* ↑, Genus: *Bacnesiellaceae* ↓, *Butyrivibrio* ↑, *Clostridiaceae 02d06* ↓, *Megasphaera* ↑, *Oscillospira* ↓, *Prevotella* ↑	SCFA concentration –
[59]	Chinese	Metformin treated for 3 days in T2DM (*n* = 22)	versus before metformin treatment in T2DM (*n* = 22) Genus: *Bacteroides* ↓ Species: *Bacteroides fragilis* ↓, *Bacteroides finegoldii* ↓, *Bacteroides thetaiotaomicron* ↓, *Bacteroides uniformis* ↓, *Bacteroides ovatus* ↓, *Bacteroides intestinalis* ↓, *Bacteroides stercoris* ↓, *Bacteroides eggerthii* ↓, *Bacteroides fluxus* ↓, *Bacteroides caccae* ↓, *Bacteroides dorei* ↓	GUDCA, Tauroursodeoxycholic acid, Conjugated Secondary bile acids ↑ Total bile acids –
**Metformin Treatment Effects in Healthy Subjects**				
[60]	Caucasian	Metformin treated for 7 days in healthy subjects (*n* = 18)	versus before metformin treatment in healthy subjects (*n* = 18) α-diversity (Shannon index): ↓ Class: Bacilli ↑, Enterobacteriales ↑, Episilonproteobacteria ↑, Gammaproteobacteria ↑, Negativicutes ↓ Order: Clostridiaceae_1 ↓, Lactobacillales ↑, Peptostreptococcaceae ↓, Selenomonadales ↓ Family: *Asaccharospora* ↓, *Enterobacteriaceae* ↑, *Romboutsia* ↓ Genus: *Blautia* ↑, *Ruminiclostridium_6* ↓, *Streptococcus* ↑	NA
[61]	Danish	Metformin treated for 6 weeks in healthy subjects (*n* = 22)	versus before metformin treatment in healthy subjects (*n* = 18) Genus: *Bilophila* ↑, *Caproiciproducens* ↑, *Clostridium_sensu_stricto_1* ↓, *Escherichia-Shigella* ↑, *Intestinibacter* ↓, *Prevotella* ↑, *Terrisporobacter* ↓ Species: *Alistipes finegoldii* ↑, *Bilophila wadsworthia* ↑, *Intestinibacter bartlettii* ↓	NA

**Table 3:** Enrolled clinical studies to investigate the relationship between the gut microbiome and metformin in recruiting or active state.

Clinical Trials.gov Identifier	Study Title	Country	Study Population	Interventions
NCT04194515	Gut Microbiota and Bile Acids in Type 2 Diabetes Mellitus	Taiwan	Outpatients and treatment-naïve male patients with type 2 diabetes	Drug: YH1 Drug: metformin
NCT04287387	Response of Gut Microbiota in Type 2 Diabetes to Hypoglycemic Agents	China	Type 2 diabetes patients (18–65 years)	Drug: Glucophage 500 mg Tablet Drug: Acarbose Tablets Drug: Sitagliptin tablet Drug: Dapagliflozin Tablet Drug: Pioglitazone Tablets Drug: Glimepiride Tablets
NCT04639492	Postbiotic MBS and Metformin Combination in Patients With T2DM	Taiwan	Type 2 diabetes patients (20–70 years)	Dietary Supplement: MBS oral solution Oral BIDAC, twice a day before breakfast and dinner times
NCT02960659	Title: Therapeutic Targets in African-American Youth With Type 2 Diabetes	United States	African-American (12–25 years)	Drug: Metformin and Liraglutide Drug: Metformin
NCT03558867	Personalized Medicine in Pre-diabetes and Early Type 2 Diabetes	Australia	Pre-diabetes or newly-diagnosed with type 2 diabetes (in the last 6 months)	Drug: Metformin + Healthy diet Drug: Metformin + Personalized diet
NCT03732690	The Interaction Between Protein Intake, Gut Microbiota and Type 2 Diabetes in Subjects With Different Ethnic Backgrounds	France	T2DM patients: Caucasian (*n* = 80), Caribbean (*n* = 40) stable dose of metformin and do not use insulin or proton-pump inhibitors.	Other: Diet HP Other: Diet LP
NCT04089280	Probiotics in Metformin Intolerant Patients With Type 2 Diabetes	Poland	T2DM patients (18–75 years) with metformin treatment in the last 3 months (<1500 mg/d)	Dietary Supplement: Sanprobi Barrier-multispecies probiotic Other: Placebo Comparator
NCT03718715	The Interaction Between Metformin and Microbiota—The MEMO Study. (MEMO)	Sweden	Newly diagnosed patients with type 2 diabetes without previous treatment with metformin (40–80 years).	Drug: Metformin
NCT03489317	Gut Microbiomes in Patients With Metabolic Syndrome	Hongkong	Residents in Hongkong (no metabolic syndrome, metabolic syndrome-partial, metabolic syndrome-full)	Drug: Metformin Behavioral: lifestyle modification Drug: Simvastatin 10 mg Drug: Amlodipine 5 mg
NCT02609815	Initial Combination of Gemigliptin and Metformin on Microbiota Change	Republic of Korea	Type 2 patients with drug naive for 6 weeks	Drug: gemigliptin/metformin Drug: glimepiride/metformin
NCT04341571	Effect of Probiotics Versus Metformin on Glycemic Control, Insulin Sensitivity and Insulin Secretion in Prediabetes.	Mexico	Pre-diabetes	Dietary Supplement: Probiotics Drug: Metformin
NCT04209075	Prebiotics and Metformin Improve Gut and Hormones in Type 2 Diabetes in Youth (MIGHTY-fiber)	United States	Type 2 patients (10–25 years)	Dietary Supplement: Biomebliss Drug: Metformin Dietary Supplement: Placebo

## Data Availability

Not applicable.
